# Mesopelagic fishes of the North-West African Upwelling from the Discovery Collections

**DOI:** 10.3897/BDJ.11.e105921

**Published:** 2023-08-09

**Authors:** Jethro George Jack Reading, Tammy Horton

**Affiliations:** 1 University of Southampton, Southampton, United Kingdom University of Southampton Southampton United Kingdom; 2 National Oceanography Centre, Southampton, United Kingdom National Oceanography Centre Southampton United Kingdom

**Keywords:** deep-sea, fishes, Mauritania, midwater

## Abstract

**Background:**

Mesopelagic fish specimens from two stations in the NW African Upwelling were identified and catalogued to produce a Darwin Core-aligned dataset. A total of 9655 individual fishes were identified, with 9017 specimens identified at least to genus level and 3124 specimens identified to species level. This dataset comprises specimens collected from the 1990 RRS *Discovery* (III) Cruise D195 and was used to investigate depth-related trends in diversity and community composition alongside species-specific migratory behaviour. The finalised dataset was published on OBIS through the Deep-Sea node.

**New information:**

This dataset contains occurrence and abundance data for midwater fishes caught between the Mauritanian coast and Cape Verde, published for the first time. The dataset records 146 different fish taxa. Twenty-three taxa in the dataset are not present in any prior OBIS datasets that cover the area. These novel taxa are: *Bathylagusandriashevi, Bolinichthysindicus, Bolinichthyssupralateralis, Cyclothoneparapallida, Dolichopteroidesbinocularis, Gigantactis* indet. *Gymnoscopelus* stet., *Howellaatlantica, Hygophumproximum, Hygophumtaaningi, Ichthyococcus polli, Lampadenaanomala, Lampanyctuscuprarius, Lampanyctusisaacsi, Lampanyctuslineatus, Lampanyctusmacdonaldi, Lampanyctusnobilis, Lestidiopsmirabilis*, *Loweinarara*, *Macroparalepisbrevis, Melamphaesmicrops* and *Melanonusgracilis*. An anglerfish specimen belonging to Linophrynidae was also found, the first in the leftvent family to be logged in the area on OBIS; however, the specimen was too damaged to identify beyond this level.

## Introduction

### Mesopelagic Fish Communities

The mesopelagic zone (200-1000 m water depth) is one of the Earth's largest habitats by volume, hosting a diverse community of fishes that globally masses between 1 and 20 gigatonnes (Proud et al. 2018). Mesopelagic fishes are of scientific interest in part due to the high degree of convergent evolution apparent across taxonomic orders, influenced by selection pressures particular to their midwater habitat (Helfman et al. 2009). This includes adaptations such as cylindrical, binocular eyes, which allow improved light-gathering ability (Priede 2017) and stereoscopic vision in dim light, which are found in at least 12 different genera across wide taxonomic distances, including opisthoproctid barreleye fishes, such as *Monacoa* Whitley 1943 and *Macropinna Chapman 1939*, *Gigantura Brauer 1901* telescope fishes, and the tube-eye fish *Stylephoruschordatus* Shaw 1791 (Collin et al. 1997). Other widespread adaptations include bioluminescence, which is used as a lure by fishes such as saccopharyngiform eels (Denton and Marshall 2009), stomiid dragonfish (Sutton and Hopkins 1996) and ceratioid anglerfish (Munk 1999, Pietsch 2009), and as counterillumination, which is found in many taxa (Davis et al. 2020a). Some species also display ultra-black skin, able to absorb as much as 99.5% of incident light, to prevent reflected ambient or bioluminescent light from giving away their position to other mesopelagic animals (Davis et al. 2020b).

Mesopelagic fishes play a key role in modulating the transfer of carbon and nutrients from the epipelagic (0-200 m) to the bathypelagic (1000-4000 m) zone, with diel vertical migration enhancing transport efficiency by bypassing the depths where passively sinking particulate organic carbon is most often remineralised (Boyd et al. 2019). Alongside this biogeochemical importance, mesopelagic fishes are of increasing interest to fisheries (Standal and Grimaldo 2021), with some authors suggesting that exploiting mesopelagic resources for fishmeal could provide as much as 4.6 kg of food per person per day globally via aquaculture (St. John et al. 2016). At present, however, global landings of mesopelagic fish are limited, with only 2.68 million tonnes landed in the last seven decades (Pauly et al. 2021), insignificant when compared to total global landings by capture fisheries that mass in the tens of millions of tonnes per year. Despite their importance, midwater ecosystems have historically been under-studied and have, therefore, been recognised as a priority for scientists in the ongoing UN Decade of Ocean Science, with improved understanding of diversity and biogeography highlighted as a key target (Howell et al. 2020). If mesopelagic fish communities are to be exploited sustainably while safeguarding their biogeochemical functions, improved knowledge of their distributions is necessary. In particular, this research should be focused on high-productivity upwelling ecosystems as these are amongst the first to have been targeted by developing mesopelagic fisheries (Pauly et al. 2021).

### The North-West African EBUE

Eastern Boundary Upwelling Ecosystems (EBUEs) are highly-productive marine ecosystems found at the eastern edges of oceanic gyres (Smith 1995). In these areas, equatorward winds interact with continental coasts and, through Ekman pumping, draw up nutrient-rich waters from the deep ocean. EBUEs support large populations of epipelagic forage fish that are already essential to fisheries, supplying 20% of global landings between 2000 and 2007 despite encompassing just 1% of the ocean by surface area (Chavez and Messié 2009). Of the four major EBUEs in the world ocean, the NW African EBUE is the least well studied, due in part to the absence of nearby oceanographic institutions (Chavez and Messié 2009). This is despite the importance of the area to food security, with 5.5 million tonnes (6.5% of global landings) of fish landed in the Eastern Central Atlantic in 2018 alone (UN FAO 2020).

Upwelling is semi-continuous between 10 and 20°N off the coast of Africa (Chavez and Messié 2009), with productivity further enhanced by allochthonous input of iron-rich Saharan dust (Pradhan et al. 2006). The Mauritanian Upwelling Ecoregion was first described as a discrete mesopelagic biogeographic area in 1977, based in part on the presence of two apparently endemic myctophids (Nafpaktitis et al. 1977) which were later found elsewhere. Later work recognised a split in the ichthyoplankton community north and south of the convergence between the Mauritanian and Cape Verde currents (John and Zelck 1997), with southerly waters dominated by the phosichthyid *Vinciguerrianimbaria* (Jordan & Williams in Jordan and Starks 1896) and northerly waters dominated by the gonostomatid *Cyclothonebraueri Jespersen and Tåning 1926*. Most recently, a global review of mesopelagic biogeography by Sutton et al. (2017) defined the Mauritania/Cape Verde ecoregion (MCV) by the presence of cold-water relict fauna and pseudoceanic species associated with African continental slopes, alongside elevated species richness. Here, the split is defined in terms of water masses, with southern upwelling originating from older, higher-nutrient South Atlantic Central Water and northern upwelling originating from younger North Atlantic Central Water. While key work on mesopelagic fishes in the broader area was conducted aboard the 1965 SOND Cruise (Badcock 1970), trawls were only conducted in the northern MCV. Here, we hope to extend knowledge of mesopelagic fish community composition into the southern MCV, by identifying and cataloguing samples collected between Cape Verde and Mauritania that are currently held in the Discovery Collections at the National Oceanography Centre, Southampton, UK.

### The Discovery Collections

The Discovery Collections are an internationally important repository of deep-sea marine benthic and pelagic invertebrate and fish specimens which contain valuable samples obtained from the global ocean since 1925. They are split into two parts (modern and historic). The modern Discovery Collections at the National Oceanography Centre (DISCOLL, NOC, UK; http://grscicoll.org/institution/national-oceanography-centre-southampton; https://noc.ac.uk/facilities/discovery-collections) consist largely of benthic biological samples collected during research programmes and major environmental surveys that have taken place off the continental shelf of the UK and Ireland since the 1970s. They are housed in a climate-controlled warehouse at the National Oceanography Centre, Southampton (NOC). The historic Discovery Collections, consisting of early Southern Ocean material and much of the early North Atlantic pelagic and some benthic material, are now housed at the Natural History Museum, London (Rainbow 2005).

Notably, the Modern Discovery Collections house specimens from the Porcupine Abyssal Plain Sustained Observatory (PAP–SO), a multidisciplinary open-ocean time series site in the NE Atlantic (48°50'N 16°30'W, 4850 m water depth), one of only two abyssal time series study sites in the world (Hartman et al. 2021). Through collaboration in international sampling programmes, the Discovery Collections also contain materials from the tropical central Pacific Ocean, the Arabian Sea and from abyssal depths near the Crozet Islands in the Southern Indian Ocean. New samples are added every year from national and international research programmes. Work is ongoing to digitise the sample information from these valuable collections. While a dataset of midwater specimens from the Discovery Collections is already available through OBIS (Pugh 2000), this does not include the current specimens and contains relatively few specimens from mesopelagic waters between the Mauritanian coast and Cape Verde.

## Project description

### Title

Mesopelagic Fishes of the North-West African Upwelling from the Discovery Collections

### Personnel

Jethro Reading, Tammy Horton, James Maclaine

### Study area description

Mesopelagic and epipelagic waters of the Eastern Central Atlantic, between the coasts of Mauritania and Western Sahara and Cape Verde

## Sampling methods

### Study extent

This dataset was compiled using specimens from the 1990 RRS *Discovery* (III) Cruise D195 (Herring 1990). Samples were taken from two stations: a northern station (12181) in the Western Saharan EEZ and a southern station (12183) in the Cape Verdean EEZ. Samples were taken during the day and night between the surface and 1000 m depth.

### Sampling description

Samples were collected during Cruise D195 on the RRS *Discovery* (III), which departed from Santa Cruz, Tenerife on 05/09/1990 and arrived at Barry, South Wales on 05/10/1990 (Herring 1990). Samples were collected using a multiple rectangular midwater trawl net (RMT8+1M) (Roe and Shale 1979). The RMT8+1M system consists of three 8 m^2^ and three 1 m^2^ mouth area nets combined within one frame, with mesh sizes of 4.5 mm and 320 µm, respectively. This system allows for multiple depth-discrete samples to be obtained in succession with just one deployment and recovery of the gear. The 8 m^2^ net will retain most mesopelagic fish, but may not have sampled ichthyoplankton and fish slimmer than 4.5 mm; however, any fish incidentally taken by the 1 m^2^ net were transferred to the same preserved lot as the larger fish. Net opening was triggered by an acoustic signal. Within the dataset, the codes RMT8M/1, RMT8M/2 and RMT8M/3 are used to refer to the individual nets within the RMT8+1M system that were used to obtain each unsorted fish sample. The event during which each fish sample was taken is hereafter referred to as a sampling event.

**Figure 1.**
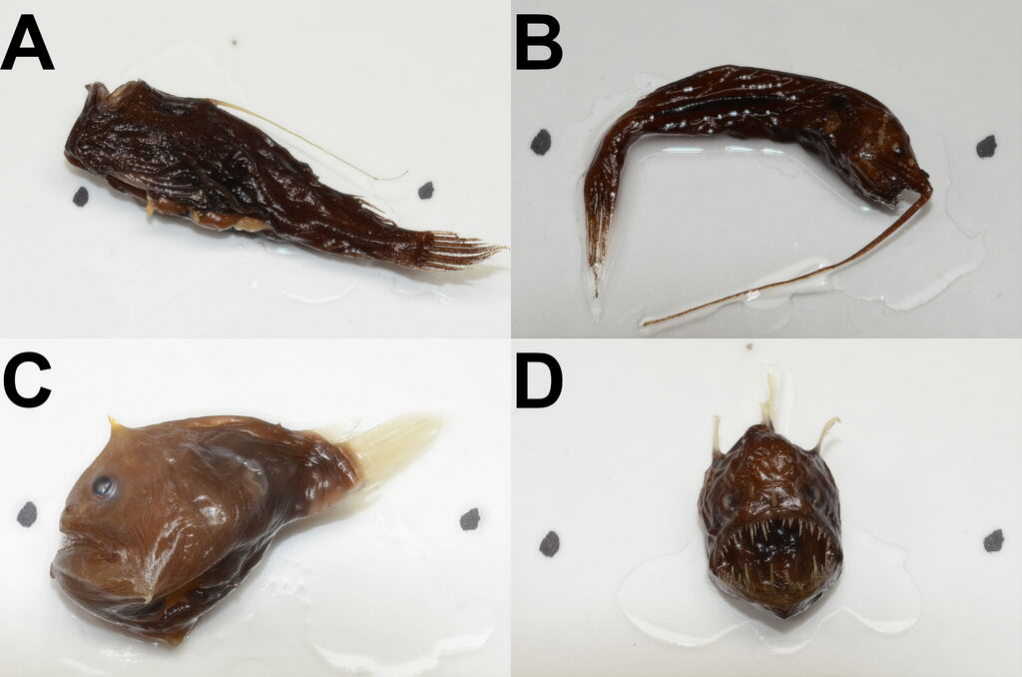
Anglerfish specimens from Cruise D195: A- *Ceratias* indet.; B- *Gigantactis* indet.; C- *Himantolophus* indet.; and D- *Melanocetusmurrayi* (Günther 1887).

Samples from Station 12183 were taken in day and night vertical series from the surface to 1000 m, with the net towed across a depth range of approximately 100 m during each sampling event. These samples were intended to quantify the biomass of zooplankton and nekton undergoing diel vertical migration and, as such, sampled organisms were volumed in bulk and not identified to species level. Sampling event 12181_4 used a net equipped with a closing cod-end and lights were attached and turned on for events 12181_12 and 12181_18. In events where the cod-end was used, the suffix CCE is added to the code (RMT8CCE), while an L has been inserted into the gear code for sampling events where lights were used and switched on (e.g. RMT8ML/3). It is worth noting that D195 was primarily conducted to investigate mesopelagic photobiology. As such, the eyes of certain taxa and the taxonomically-diagnostic lures and caruncles of ceratioid anglerfish (Fig. 1) were often removed, alongside certain whole individuals frozen for genetic analysis. As a result, most anglerfish could not be identified to species level, with the exception of members of the genus *Melanocetus* which could be distinguished by vomerine teeth.

Unfortunately, using available resources, it has been impossible to discern the exact number and identity of specimens removed. A survey of the ichthyology collections at the Natural History Museum, London, did, however, reveal a number of other specimens from Cruise D195, including both previously identified and accessioned specimens and unidentified fishes. These specimens have not been included in the current dataset, which represents only those fishes held in the Discovery Collections in Southampton. Additional samples from further *RRS Discovery* Cruises in the Mauritanian Upwelling held in the Discovery Collections at Southampton may be added to this dataset in the future. To enable researchers to locate fish specimens from D195 stored at the NHM, a search string for the NHM Data Portal was created (https://doi.org/10.5519/qd.1rb61gc6). This search string will access records of both specimens already identified and also any specimens from the cruise accessioned in the future.

## Geographic coverage

### Description

**Figure 2.**
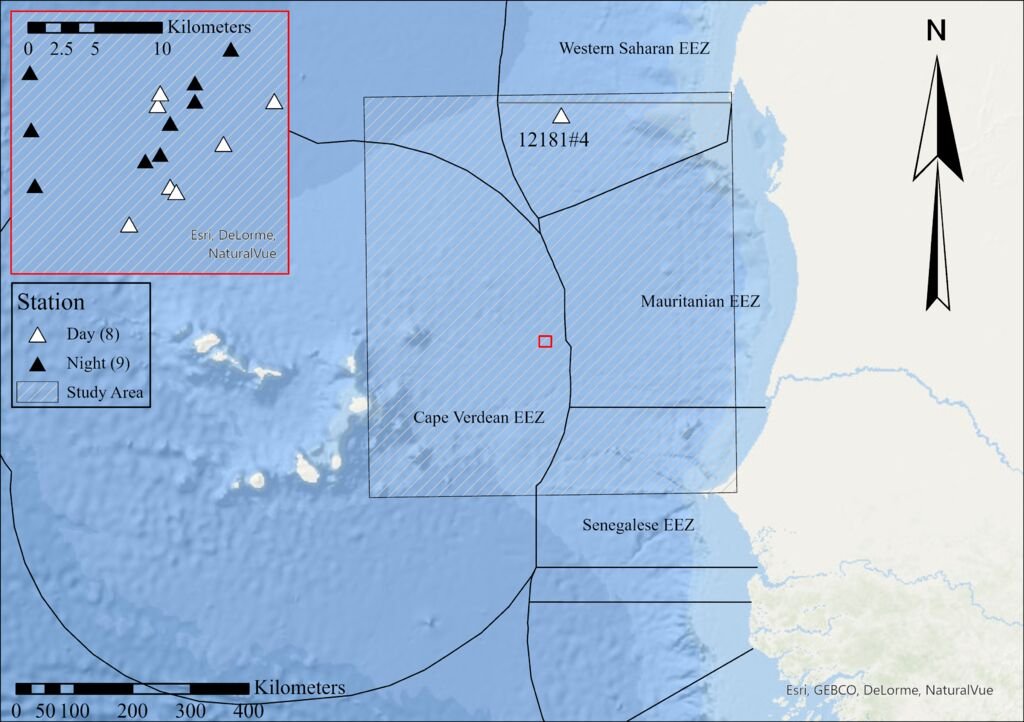
Sample location map: The single sampling event (12181#4) from the Northern Station, located within the EEZ of Western Sahara, is shown on the wider-scale map. All sampling events from the Southern Station (12183), located within the EEZ of Cape Verde, are plotted inside the red inset. Daytime samples are indicated with a white triangle, night-time trawls with a black triangle. The Study Area polygon, used to identify novel local records, is shown by the grey-hatched rectangle. Map produced in ArcGIS.

Samples were collected from two stations in the open ocean between Mauritania and Cape Verde, with a northern station located in the EEZ of Western Sahara and a southern station located in the Cape Verdean EEZ. The study area was defined as a rectangle (Fig. 2), with its north-eastern corner located at Nouadhibou, Mauritius, southernmost extent level with Dakar, Senegal and westernmost extent level with the eastern coast of Boa Vista, Cape Verde. This study area was defined by the first author using these borders so that it covered bathyal depths between Mauritania and Cape Verde, thereby encompassing much of the mesopelagic ecosystem influenced by the Mauritanian Upwelling. This study area was used to later compare our new occurrence data with existing OBIS records. Samples were taken from the epipelagic (5 m) to the deepest part of the mesopelagic (1000 m). The high abundance of the phosichthyid *Vinciguerrianimbaria* (Jordan & Williams, 1895) and scarcity of the gonostomatid *Cyclothonebraueri* Jespersen & Tåning, 1926 in shallower (0-400 m) samples from the southern station suggests that these samples were taken from south of the Cape Verde Frontal Zone, as differing abundances of these species have been found either side of this biogeographic barrier (John and Zelck 1997). Samples from the depths where *C.braueri* is most often found (200-900 m) were not available from the northern station (Froese and Pauly 2022).

### Coordinates

14.711 and 20.841 Latitude; -22.669W and -17.004 Longitude.

## Taxonomic coverage

### Description

**Figure 3.**
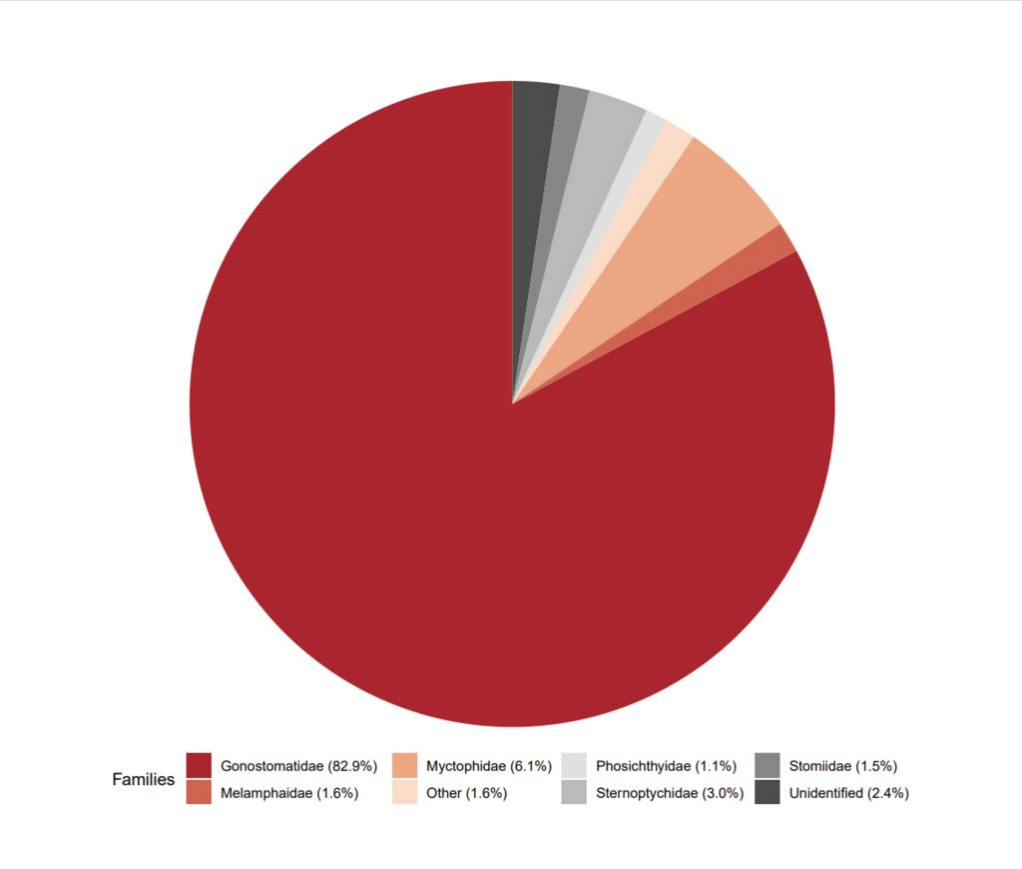
Pie chart showing numbers of individual fishes identified by family. Here, Unidentified refers to specimens that could not be identified to family level, either due to damage or due to larval state. Other refers to specimens that belong to families that did not make up more than 1% of total fish abundance. Percentage of total fish abundance have been given for each family listed.

A total of 146 taxa were identified, all belonging to the class Actinopterygii. Table 1 contains full taxonomic placements of all taxa in the dataset, alongside the total number of specimens of each taxon across the dataset (Total Abundance) and the number of times each taxon was present in the sampling events (Occurrences). The primary guide used to identify fish was the recently published UN FAO *Identification guide to the mesopelagic fishes of the central and south east Atlantic Ocean* (Sutton et al. 2020). Ceratioids belonging to families not covered in this guide were identified using taxonomic keys available from ToLWeb (Pietsch and Kenaley 2007). Species-level identification of anglerfish in the genus *Melanocetus* Günther 1864 was further aided by illustrations kindly provided by Theodore Pietsch (Pietsch and Van Duzer 1980). Where necessary, Open Nomenclature qualifiers have been added to taxon names to indicate uncertainty (Sigovini et al. 2016, Horton et al. 2021). Taxa that are newly recorded from the study area in OBIS are marked with an asterisk in Table 1. Twenty-seven families were identified Fig. 3, with the most abundant families (Gonostomatidae, Myctophidae, Sternoptychidae, Melamphaiidae, Stomiidae and Phosichthyidae) contributing over 95% of individuals, with no other families represented by > 100 specimens. Taxonomy and authority are taken from WoRMS (WoRMS Editorial Board 2023) and common names, where given, are taken from FishBase (Froese and Pauly 2022). Fish are arranged by order according to the phylogeny found in Betancur-R et al. (2017).

## Temporal coverage

**Data range:** 1990-9-13 – 1990-9-21.

### Notes

All samples were collected in the above time period, but were sorted and identified in between July 2021 and May 2022.

## Collection data

### Collection name

Discovery Collections

### Collection identifier

DISCOLL

### Specimen preservation method

4% borax-buffered formaldehyde, transferred to 80% ethanol during identification process.

## Usage licence

### Usage licence

Creative Commons Public Domain Waiver (CC-Zero)

## Data resources

### Data package title

Mauritanian Midwater fish in the Discovery Collections

### Resource link

http://ipt.iobis.org/obis-deepsea/resource?r=midwater_fish_mauritania

### Number of data sets

1

### Data set 1.

#### Data set name

Mauritanian Midwater fish in the Discovery Collections

#### Data format

Darwin Core

#### Download URL

https://obis.org/dataset/055a97b8-3a3a-4f68-8bbe-f9dab0646d32

#### Description

This dataset (Reading and Horton 2023) contains occurrence and abundance information on mesopelagic fishes sampled during Cruise D195 of the RRS *Discovery* (III), which was initially conducted to investigate the photobiology of mesopelagic organisms. Fishes were held unsorted in the Discovery Collections, at the National Oceanography Centre, Southampton for 30 years before being sorted, identified and digitised as part of J. Reading's MSci project at the University of Southampton.

**Data set 1.** 

Column label	Column description
parentEventID	An identifier for the broader Event that groups this and potentially other Events (http://rs.tdwg.org/dwc/terms/parentEventID).
verbatimEventDate	The verbatim original representation of the date and time information for an Event (http://rs.tdwg.org/dwc/terms/verbatimEventDate).
InstitutionID	An identifier for the institution having custody of the object(s) or information referred to in the record (http://rs.tdwg.org/dwc/terms/institutionID).
InstitutionCode	The name (or acronym) in use by the institution having custody of the object(s) or information referred to in the record (http://rs.tdwg.org/dwc/terms/institutionCode).
waterBody	The name of the water body in which the Location occurs (http://rs.tdwg.org/dwc/terms/waterBody).
eventID	An identifier for the set of information associated with an Event (something that occurs at a place and time). May be a global unique identifier or an identifier specific to the data set (http://rs.tdwg.org/dwc/terms/eventID).
fieldNumber	An identifier given to the event in the field. Often serves as a link between field notes and the Event (http://rs.tdwg.org/dwc/iri/fieldNumber).
eventDate	The date-time or interval during which an Event occurred. For occurrences, this is the date-time when the event was recorded. Not suitable for a time in a geological context (http://rs.tdwg.org/dwc/terms/eventDate).
verbatimDepth	The original description of the depth below the local surface (http://rs.tdwg.org/dwc/terms/verbatimDepth).
habitat	A category or description of the habitat in which the Event occurred (http://rs.tdwg.org/dwc/terms/habitat).
higherGeography	A list (concatenated and separated) of geographic names less specific than the information captured in the locality term (http://rs.tdwg.org/dwc/terms/higherGeography).
higherGeographyID	Recommended best practice is to use a persistent identifier from a controlled vocabulary such as the Getty Thesaurus of Geographic Names (http://rs.tdwg.org/dwc/terms/higherGeographyID).
Locality	The specific description of the place (http://rs.tdwg.org/dwc/terms/locality).
locationID	An identifier for the set of location information (data associated with dcterms:Location). May be a global unique identifier or an identifier specific to the data set (http://rs.tdwg.org/dwc/terms/locationID).
minimumDepthInMeters	The lesser depth of a range of depth below the local surface, in meters (http://rs.tdwg.org/dwc/terms/minimumDepthInMeters).
maximumDepthInMeteres	The greater depth of a range of depth below the local surface, in meters (http://rs.tdwg.org/dwc/terms/maximumDepthInMeters).
verbatimDepth	The original description of the depth below the local surface (http://rs.tdwg.org/dwc/terms/verbatimDepth).
verbatimCoordinates	The verbatim original spatial coordinates of the Location. The coordinate ellipsoid, geodeticDatum, or full Spatial Reference System (SRS) for these coordinates should be stored in verbatimSRS and the coordinate system should be stored in verbatimCoordinateSystem (http://rs.tdwg.org/dwc/terms/verbatimCoordinates).
verbatimCoordinateSystem	The spatial coordinate system for the verbatimLatitude and verbatimLongitude or the verbatimCoordinates of the Location (http://rs.tdwg.org/dwc/iri/verbatimCoordinateSystem).
VerbatimSRS	The ellipsoid, geodetic datum, or spatial reference system (SRS) upon which coordinates given in verbatimLatitude and verbatimLongitude, or verbatimCoordinates are based (http://rs.tdwg.org/dwc/terms/verbatimSRS).
decimalLatitude	The geographic latitude (in decimal degrees, using the spatial reference system given in geodeticDatum) of the geographic center of a Location. Positive values are north of the Equator, negative values are south of it. Legal values lie between -90 and 90, inclusive (http://rs.tdwg.org/dwc/terms/decimalLatitude).
decimalLongitude	The geographic longitude (in decimal degrees, using the spatial reference system given in geodeticDatum) of the geographic center of a Location. Positive values are east of the Greenwich Meridian, negative values are west of it. Legal values lie between -180 and 180, inclusive (http://rs.tdwg.org/dwc/terms/decimalLongitude).
geodeticDatum	The ellipsoid, geodetic datum, or spatial reference system (SRS) upon which the geographic coordinates given in decimalLatitude and decimalLongitude are based (http://rs.tdwg.org/dwc/terms/geodeticDatum).
footprintWKT	A Well-Known Text (WKT) representation of the shape (footprint, geometry) that defines the Location. A Location may have both a point-radius representation (see decimalLatitude) and a footprint representation, and they may differ from each other (http://rs.tdwg.org/dwc/terms/footprintWKT).
footprintSRS	The ellipsoid, geodetic datum, or spatial reference system (SRS) upon which the geometry given in footprintWKT is based (http://rs.tdwg.org/dwc/terms/footprintSRS).
coordinateUncertaintyinMeters	The horizontal distance (in meters) from the given decimalLatitude and decimalLongitude describing the smallest circle containing the whole of the Location. Leave the value empty if the uncertainty is unknown, cannot be estimated, or is not applicable (because there are no coordinates). Zero is not a valid value for this term (http://rs.tdwg.org/dwc/terms/coordinateUncertaintyInMeters).
coordinateprecision	A decimal representation of the precision of the coordinates given in the decimalLatitude and decimalLongitude (http://rs.tdwg.org/dwc/terms/coordinatePrecision).
eventRemarks	Comments or notes about the Event (http://rs.tdwg.org/dwc/terms/eventRemarks).
fieldNotes	One of a) an indicator of the existence of, b) a reference to (publication, URI), or c) the text of notes taken in the field about the Event (http://rs.tdwg.org/dwc/terms/fieldNotes).
samplingProtocol	The names of, references to, or descriptions of the methods or protocols used during an Event (http://rs.tdwg.org/dwc/terms/samplingProtocol).
measurementType	The nature of the measurement, fact, characteristic, or assertion (http://rs.tdwg.org/dwc/terms/version/measurementType-2018-09-06).
measurementTypeID	NVS ID for nature of measurement.
occurrenceID	An identifier for the Occurrence (as opposed to a particular digital record of the occurrence). In the absence of a persistent global unique identifier, construct one from a combination of identifiers in the record that will most closely make the occurrenceID globally unique (http://rs.tdwg.org/dwc/terms/occurrenceID).
measurementValue	The value of the measurement, fact, characteristic, or assertion (http://rs.tdwg.org/dwc/terms/measurementValue).
measurementValueID	NVS ID for measurement value.
measurementUnit	The units associated with the measurementValue (http://rs.tdwg.org/dwc/terms/measurementUnit).
measurementUnitID	NVS ID for unit of measurement.
measurementDeterminedDate	The date on which the MeasurementOrFact was made (http://rs.tdwg.org/dwc/terms/measurementDeterminedDate).
measurementDeterminedBy	A list (concatenated and separated) of names of people, groups, or organizations who determined the value of the MeasurementOrFact (http://rs.tdwg.org/dwc/terms/measurementDeterminedBy).
measurementRemarks	Comments or notes accompanying the MeasurementOrFact (http://rs.tdwg.org/dwc/terms/measurementRemarks).
language	Alanguage of the resource (http://purl.org/dc/elements/1.1/language).
collectionCode	The name, acronym, coden, or initialism identifying the collection or data set from which the record was derived (http://rs.tdwg.org/dwc/terms/collectionCode).
datasetName	The name identifying the data set from which the record was derived (http://rs.tdwg.org/dwc/terms/datasetName).
basisOfRecord	The specific nature of the data record (http://rs.tdwg.org/dwc/terms/basisOfRecord).
recordedBy	A list (concatenated and separated) of names of people, groups, or organizations responsible for recording the original Occurrence. The primary collector or observer, especially one who applies a personal identifier (recordNumber), should be listed first (http://rs.tdwg.org/dwc/terms/recordedBy).
occurrenceStatus	A statement about the presence or absence of a Taxon at a Location (http://rs.tdwg.org/dwc/terms/version/occurrenceStatus-2021-07-15).
preparations	A list (concatenated and separated) of preparations and preservation methods for a specimen (http://rs.tdwg.org/dwc/terms/preparations).
identifiedBy	A list (concatenated and separated) of names of people, groups, or organizations who assigned the Taxon to the subject (http://rs.tdwg.org/dwc/terms/identifiedBy).
dateIdentified	The date on which the subject was determined as representing the Taxon (http://rs.tdwg.org/dwc/terms/dateIdentified).
typeStatus	A list (concatenated and separated) of nomenclatural types (type status, typified scientific name, publication) applied to the subject (http://rs.tdwg.org/dwc/terms/typeStatus).
scientificNameID	An identifier for the nomenclatural (not taxonomic) details of a scientific name (http://rs.tdwg.org/dwc/terms/scientificNameID).
taxonConceptID	An identifier for the taxonomic concept to which the record refers - not for the nomenclatural details of a taxon (http://rs.tdwg.org/dwc/terms/taxonConceptID).
scientificName	The full scientific name, with authorship and date information if known. When forming part of an Identification, this should be the name in lowest level taxonomic rank that can be determined. This term should not contain identification qualifications, which should instead be supplied in the IdentificationQualifier term (http://rs.tdwg.org/dwc/terms/scientificName).
datasetID	An identifier for the set of data. May be a global unique identifier or an identifier specific to a collection or institution (http://rs.tdwg.org/dwc/terms/datasetID).
identificationRemarks	Comments or notes about the Identification (http://rs.tdwg.org/dwc/terms/identificationRemarks).
identificationQualifier	A controlled value to express the determiner's doubts about the Identification (http://rs.tdwg.org/dwc/iri/identificationQualifier).
kingdom	The full scientific name of the kingdom in which the taxon is classified (http://rs.tdwg.org/dwc/terms/kingdom).
phylum	The full scientific name of the phylum or division in which the taxon is classified (http://rs.tdwg.org/dwc/terms/phylum).
class	The full scientific name of the class in which the taxon is classified (http://rs.tdwg.org/dwc/terms/class).
order	The full scientific name of the order in which the taxon is classified (http://rs.tdwg.org/dwc/terms/order).
family	The full scientific name of the family in which the taxon is classified (http://rs.tdwg.org/dwc/terms/family).
genus	The full scientific name of the genus in which the taxon is classified (http://rs.tdwg.org/dwc/terms/genus).
subgenus	The full scientific name of the subgenus in which the taxon is classified. Values should include the genus to avoid homonym confusion (http://rs.tdwg.org/dwc/terms/subgenus).
specificEpithet	The name of the first or species epithet of the scientificName (http://rs.tdwg.org/dwc/terms/specificEpithet).
infraspecificEpithet	The name of the first or species epithet of the scientificName (http://rs.tdwg.org/dwc/terms/specificEpithet).
taxonRank	The taxonomic rank of the most specific name in the scientificName (http://rs.tdwg.org/dwc/terms/taxonRank).
scientificNameAuthorship	The authorship information for the scientificName formatted according to the conventions of the applicable nomenclaturalCode (http://rs.tdwg.org/dwc/terms/scientificNameAuthorship).

## Figures and Tables

**Table 1.** Classification, abundance and number of occurrences for every taxon in the Mauritanian dataset. Taxa newly recorded on OBIS in the study area are marked with an asterisk. Here, Scientific Name is used to indicate the lowest taxonomic rank to which specimens were identified. Where possible, common names are provided and the applicable ON qualifiers as per Sigovini et al. (2016) are given where necessary. Higher-level classification is in phylogenetic order, while, within orders, families, genera and species are alphabetised.

Order	Family	Genus	Species Name	ON Qualifier	Common Name	Total Abundance	Occurrences
N/A			Actinopterygii	indet.	Ray-finned Fishes	198	15
N/A			Actinopterygii	stet.	Ray-finned Fishes	5	3
Anguilliformes			sp.	stet.	Eels	5	3
Anguilliformes	Derichthyidae	*Nessorhamphus* Schmidt, 1931	*N.ingolfianus* (Schmidt, 1912)		Duckbill Oceanic Eel	1	1
Anguilliformes	Nemichthyidae		sp.	indet.	Snipe eels	4	2
Anguilliformes	Nemichthyidae	*Nemichthys* Richardson, 1848	*N.curvirostris* (Strömman, 1896)		Pale Threadtail Snipe Eel	1	1
Anguilliformes	Nemichthyidae	*Nemichthys* Richardson, 1848	*N.scolopaceus* Richardson, 1848		Slender Snipe Eel	1	1
Anguilliformes	Serrivomeridae	*Serrivomer* Gill & Ryder, 1883	*Serrivomer* sp.	stet.	Sawtooth eels	6	5
Saccopharyngiformes	Eurypharyngidae	*Eurypharynx* Vaillant, 1882	*E.pelecanoides* Vaillant, 1882		Pelican Eel	2	1
Alepocephaliformes	Platytroctidae		sp.	stet.	Tubeshoulders	9	3
Alepocephaliformes	Platytroctidae	*Holtbyrnia* Parr, 1937	*Holtbyrnia* sp.	stet.		2	1
Alepocephaliformes	Platytroctidae	*Searsia* Parr, 1937	*S.koefoedi* Parr, 1937		Koefoed's Searsid	9	5
Argentiniformes	Bathylagidae		sp.	indet.	Deep-sea smelts	1	1
Argentiniformes	Bathylagidae	*Bathylagichthys* Kobyliansky, 1986	*Bathylagichthys* sp.	indet.		1	2
Argentiniformes	Bathylagidae	*Bathylagichthys* Kobyliansky, 1986	*Bathylagichthys* sp.	stet.		2	4
Argentiniformes	Bathylagidae	*Bathylagoides* Whitley, 1951	*B.argyrogaster* (Norman, 1930)		Silver deep-sea smelt	12	5
Argentiniformes	Bathylagidae	*Bathylagus* Günther, 1878	*B.andriashevi* Kobyliansky, 1986 *			1	1
Argentiniformes	Bathylagidae	*Bathylagus* Günther, 1878	*B.euryops* Goode & Bean, 1896		Goiter blacksmelt	20	2
Argentiniformes	Bathylagidae	*Melanolagus* Kobyliansky, 1986	*M.bericoides* (Borodin, 1929)		Bigscale Deepsea Smelt	1	1
Argentiniformes	Opisthoproctidae	*Dolichopteroides* Parin, Belyanina & Evseenko, 2009	*D.binocularis* (Beebe, 1932) *			1	1
Argentiniformes	Opisthoproctidae	*Opisthoproctus* Vaillant, 1888	*O.soleatus* Vaillant, 1888		Barrel-eye	2	1
Stomiiformes	Gonostomatidae	*Bonapartia* Goode & Bean, 1896	*B.pedaliota* Goode & Bean, 1896		Longray fangjaw	1	1
Stomiiformes	Gonostomatidae	*Cyclothone* Goode & Bean, 1883	*Cyclothone* sp.	indet.	Bristlemouths	5616	12
Stomiiformes	Gonostomatidae	*Cyclothone* Goode & Bean, 1883	*Cyclothone* sp.	stet.	Bristlemouths	138	3
Stomiiformes	Gonostomatidae	*Cyclothone* Goode & Bean, 1883	*C.acclinidens* Garman, 1899		Bentjaw bristlemouth	340	8
Stomiiformes	Gonostomatidae	*Cyclothone* Goode & Bean, 1883	*C.alba* Brauer, 1906			47	2
Stomiiformes	Gonostomatidae	*Cyclothone* Goode & Bean, 1883	*C.braueri* Jespersen & Tåning, 1926		Garrick	7	1
Stomiiformes	Gonostomatidae	*Cyclothone* Goode & Bean, 1883	*C.livida* Brauer, 1902			1090	10
Stomiiformes	Gonostomatidae	*Cyclothone* Goode & Bean, 1883	*C.pallida* Brauer, 1902		Tan bristlemouth	711	8
Stomiiformes	Gonostomatidae	*Cyclothone* Goode & Bean, 1883	*C.parapallida* Badcock, 1982 *		Shadow bristlemouth	13	2
Stomiiformes	Gonostomatidae	*Cyclothone* Goode & Bean, 1883	*C.pseudopallida* Mukhacheva, 1964		Slender bristlemouth	27	2
Stomiiformes	Gonostomatidae	*Diplophos* Günther, 1873	*D.taenia* Günther, 1873 *		Pacific portholefish	1	1
Stomiiformes	Gonostomatidae	*Gonostoma* Rafinesque, 1810	*G.atlanticum* Norman, 1930		Atlantic fangjaw	2	2
Stomiiformes	Gonostomatidae	*Gonostoma* Rafinesque, 1810	*G.denudatum* Rafinesque, 1810			2	2
Stomiiformes	Gonostomatidae	*Sigmops* Gill, 1883	*S.elongatus* (Günther, 1878)		Elongated bristlemouth	5	2
Stomiiformes	Phosichthyidae	*Ichthyococcus* Bonaparte, 1840	*I.ovatus* (Cocco, 1838)			2	1
Stomiiformes	Phosichthyidae	*Ichthyococcus* Bonaparte, 1840	*I.polli* Blache, 1964 *			3	1
Stomiiformes	Phosichthyidae	*Vinciguerria* Jordan & Evermann, 1896	*Vinciguerria* sp.	stet.		1	1
Stomiiformes	Phosichthyidae	*Vinciguerria* Jordan & Evermann, 1896	*V.attenuata* (Cocco, 1838)		Slender lightfish	29	1
Stomiiformes	Phosichthyidae	*Vinciguerria* Jordan & Evermann, 1896	*V.nimbaria* (Jordan & Williams, 1895)		Oceanic lightfish	72	2
Stomiiformes	Sternoptychidae		sp.	indet.	Hatchetfish	15	2
Stomiiformes	Sternoptychidae		sp.	stet.	Hatchetfish	22	5
Stomiiformes	Sternoptychidae	*Argyropelecus* Cocco, 1829	*Argyropelecus* sp.	indet.		15	2
Stomiiformes	Sternoptychidae	*Argyropelecus* Cocco, 1829	*A.aculeatus* Valenciennes, 1850		Lovely hatchetfish	3	1
Stomiiformes	Sternoptychidae	*Argyropelecus* Cocco, 1829	*A.affinis* Garman, 1899		Pacific hatchetfish	21	4
Stomiiformes	Sternoptychidae	*Argyropelecus* Cocco, 1829	*A.hemigymnus* Cocco, 1829		Half-naked hatchetfish	64	4
Stomiiformes	Sternoptychidae	*Argyropelecus* Cocco, 1829	*A.sladeni* Regan, 1908		Sladen's hatchetfish	30	3
Stomiiformes	Sternoptychidae	*Polyipnus* Günther, 1887	*P.polli* Schultz, 1961		Round hatchetfish	11	2
Stomiiformes	Sternoptychidae	*Sternoptyx* Hermann, 1781	*Sternoptyx* sp.	indet.		8	1
Stomiiformes	Sternoptychidae	*Sternoptyx* Hermann, 1781	*S.diaphana* Hermann, 1781		Diaphanous hatchetfish	95	12
Stomiiformes	Sternoptychidae	*Sternoptyx* Hermann, 1781	*S.pseudobscura* Baird, 1971		Highlight hatchetfish	2	1
Stomiiformes	Sternoptychidae	*Valenciennellus* Jordan & Evermann, 1896	*V.tripunctulatus* (Esmark, 1871)		Constellationfish	2	2
Stomiiformes	Stomiidae		sp.	indet.	Dragonfish	93	5
Stomiiformes	Stomiidae		sp.	stet.	Dragonfish	6	1
Stomiiformes	Stomiidae	*Aristostomias* Zugmayer, 1913	*Aristostomias* sp.	stet.		1	1
Stomiiformes	Stomiidae	*Bathophilus* Giglioli, 1882	*B.vaillanti* (Zugmayer, 1911)			1	1
Stomiiformes	Stomiidae	*Borostomias* Regan, 1908	*B.elucens* (Brauer, 1906)			1	1
Stomiiformes	Stomiidae	*Chauliodus* Bloch & Schneider, 1801	*C.schmidti* Ege, 1948			19	9
Stomiiformes	Stomiidae	*Chauliodus* Bloch & Schneider, 1801	*C.sloani* Bloch & Schneider, 1801		Sloane's viperfish	1	1
Stomiiformes	Stomiidae	*Leptostomias* Gilbert, 1905	*Leptostomias* sp.	indet.		1	1
Stomiiformes	Stomiidae	*Malacosteus* Ayres, 1848	*Malacosteus* sp.	indet.		1	1
Stomiiformes	Stomiidae	*Malacosteus* Ayres, 1848	*M.niger* Ayres, 1848		Stoplight loosejaw	6	5
Stomiiformes	Stomiidae	*Pachystomias* Günther, 1887	*Pachystomias* sp.	stet.		1	1
Stomiiformes	Stomiidae	*Pachystomias* Günther, 1887	*P.microdon* (Günther, 1878)		Smalltooth dragonfish	1	1
Stomiiformes	Stomiidae	*Photonectes* Günther, 1887	*Photonectes* sp.	indet.		2	1
Stomiiformes	Stomiidae	*Photostomias* Collett, 1889	*Photostomias* sp.	stet.		1	1
Stomiiformes	Stomiidae	*Photostomias* Collett, 1889	*P.atrox* (Alcock, 1890)			1	1
Stomiiformes	Stomiidae	*Photostomias* Collett, 1889	*P.guernei* Collett, 1889			2	1
Stomiiformes	Stomiidae	*Stomias* Cuvier, 1816	*Stomias* sp.	indet.		2	1
Stomiiformes	Stomiidae	*Stomias* Cuvier, 1816	*S.boaboa* (Risso, 1810)		Boa dragonfish	1	1
Stomiiformes	Stomiidae	*Stomias* Cuvier, 1816	*S.lampropeltis* Gibbs, 1969			2	2
Aulopiformes	Omosudidae	*Omosudis* Günther, 1887	*O.lowii* Günther, 1887		Hammerjaw	1	1
Aulopiformes	Paralepididae		sp.	indet.	Barracudinas	5	3
Aulopiformes	Paralepididae	*Lestidiops* Hubbs, 1916	*Lestidiops* sp.	stet.		2	1
Aulopiformes	Paralepididae	*Lestidiops* Hubbs, 1916	*L.mirabilis* (Ege, 1933) *		Strange pike smelt	4	3
Aulopiformes	Paralepididae	*Macroparalepis* Burton, 1934	*M.brevis* Ege, 1933 *			4	3
Aulopiformes	Scopelarchidae	*Scopelarchus* Alcock, 1896	*S.guentheri* Alcock, 1896		Staring pearleye	1	1
Myctophiformes	Myctophidae			indet.	Lanternfish	214	16
Myctophiformes	Myctophidae	*Benthosema* Goode & Bean, 1896	*B.glaciale* (Reinhardt, 1837)		Glacier lanternfish	7	3
Myctophiformes	Myctophidae	*Benthosema* Goode & Bean, 1896	*B.suborbitale* (Gilbert, 1913)		Smallfin lanternfish	5	2
Myctophiformes	Myctophidae	*Bolinichthys* Paxton, 1972	*B.indicus* (Nafpaktitis & Nafpaktitis, 1969) *			1	1
Myctophiformes	Myctophidae	*Bolinichthys* Paxton, 1972	*B.supralateralis* (Parr, 1928) *		Stubby lanternfish	6	1
Myctophiformes	Myctophidae	Ceratoscopelus Günther, 1864	*Ceratoscopelus* sp.	indet.		4	1
Myctophiformes	Myctophidae	*Ceratoscopelus* Günther, 1864	*C.maderensis* (Lowe, 1839)		Madeira lanternfish	4	1
Myctophiformes	Myctophidae	*Ceratoscopelus* Günther, 1864	*C.warmingii* (Lütken, 1892)		Warming's lanternfish	30	4
Myctophiformes	Myctophidae	*Dasyscopelus* Günther, 1864	*D.asper* (Richardson, 1845)		Prickly lanternfish	1	1
Myctophiformes	Myctophidae	*Dasyscopelus* Günther, 1864	*D.obtusirostris* (Tåning, 1928)		Bluntsnout lanternfish	1	1
Myctophiformes	Myctophidae	*Diaphus* Eigenmann & Eigenmann, 1890	*D.bertelseni* Nafpaktitis, 1966		Bertelsen's lanternfish	9	1
Myctophiformes	Myctophidae	*Diaphus* Eigenmann & Eigenmann, 1890	*D.dumerilii* (Bleeker, 1856)		Dumeri's lanternfish	19	2
Myctophiformes	Myctophidae	*Diaphus* Eigenmann & Eigenmann, 1890	*D.luetkeni* (Brauer, 1904)		Luetken's lanternfish	2	2
Myctophiformes	Myctophidae	*Diaphus* Eigenmann & Eigenmann, 1890	*D.mollis* Tåning, 1928		Soft lanternfish	5	2
Myctophiformes	Myctophidae	*Diaphus* Eigenmann & Eigenmann, 1890	*D.rafinesquii* (Cocco, 1838)		White-spotted lanternfish	2	1
Myctophiformes	Myctophidae	*Diaphus* Eigenmann & Eigenmann, 1890	*D.vanhoeffeni* (Brauer, 1906)			6	2
Myctophiformes	Myctophidae	*Diogenichthys* Bolin, 1939	*D.atlanticus* (Tåning, 1928)		Longfin lanternfish	5	3
Myctophiformes	Myctophidae	*Electrona* Goode & Bean, 1896	*E.risso* (Cocco, 1829)		Electric lanternfish	2	2
Myctophiformes	Myctophidae	*Gonichthys* Gistel, 1850	*G.cocco* (Cocco, 1829)		Cocco's lanternfish	2	1
Myctophiformes	Myctophidae	*Gymnoscopelus* Günther, 1873	*Gymnoscopelus* sp. *	stet.		1	1
Myctophiformes	Myctophidae	*Hygophum* Bolin, 1939	*H.benoiti* (Cocco, 1838)		Benoit's Lanternfish	21	6
Myctophiformes	Myctophidae	*Hygophum* Bolin, 1939	*H.macrochir* (Günther, 1864)		Large-finned Lanternfish	9	3
Myctophiformes	Myctophidae	*Hygophum* Bolin, 1939	*H.proximum*Becker, 1965 *		Firefly Lanternfish	1	1
Myctophiformes	Myctophidae	*Hygophum* Bolin, 1939	*H.reinhardtii* (Lütken, 1892)		Reinhardt's lanternfish	1	1
Myctophiformes	Myctophidae	*Hygophum* Bolin, 1939	*H.taaningi* Becker, 1965 *		Tåning's lanternfish	3	1
Myctophiformes	Myctophidae	*Lampadena* Goode & Bean, 1893	*L.anomala* Parr, 1928 *		Anomalous lanternfish	4	1
Myctophiformes	Myctophidae	*Lampanyctus* Bonaparte, 1840	*Lampanyctus* sp.	indet.		1	1
Myctophiformes	Myctophidae	*Lampanyctus* Bonaparte, 1840	*L.alatus* Goode & Bean, 1896		Winged lanternfish	114	10
Myctophiformes	Myctophidae	*Lampanyctus* Bonaparte, 1840	*L.ater* Tåning, 1928			37	7
Myctophiformes	Myctophidae	*Lampanyctus* Bonaparte, 1840	*L.cuprarius* Tåning, 1928 *			1	1
Myctophiformes	Myctophidae	*Lampanyctus* Bonaparte, 1840	*L.isaacsi* Wisner, 1974 *			7	2
Myctophiformes	Myctophidae	*Lampanyctus* Bonaparte, 1840	*L.lineatus* Tåning, 1928 *			19	4
Myctophiformes	Myctophidae	*Lampanyctus* Bonaparte, 1840	*L.macdonaldi* (Goode & Bean, 1896) *		Rakery beaconlamp	8	5
Myctophiformes	Myctophidae	*Lampanyctus* Bonaparte, 1840	*L.nobilis* Tåning, 1928 *		Noble lampfish	1	1
Myctophiformes	Myctophidae	*Lampanyctus* Bonaparte, 1840	*L.photonotus* Parr, 1928			3	1
Myctophiformes	Myctophidae	*Lepidophanes* Fraser-Brunner, 1949	*Lepidophanes* sp.	indet.		1	1
Myctophiformes	Myctophidae	*Lepidophanes* Fraser-Brunner, 1949	*L.guentheri* (Goode & Bean, 1896)		Günther's lanternfish	2	1
Myctophiformes	Myctophidae	*Lobianchia* Gatti, 1904	*L.dofleini* (Zugmayer, 1911)		Doflein's lanternfish	1	1
Myctophiformes	Myctophidae	*Lobianchia* Gatti, 1904	*L.gemellarii* (Cocco, 1838)		Cocco's lanternfish	2	1
Myctophiformes	Myctophidae	*Loweina* Fowler, 1925	*L.rara* (Lütken, 1892) *		Laura's lanternfish	3	2
Myctophiformes	Myctophidae	*Myctophum* Rafinesque, 1810	*M.affine* (Lütken, 1892)		Metallic lanternfish	1	1
Myctophiformes	Myctophidae	*Notolychnus* Fraser-Brunner, 1949	*N.valdiviae* (Brauer, 1904)		Topside lanternfish	6	2
Myctophiformes	Myctophidae	*Notoscopelus* Günther, 1864	*N.resplendens* (Richardson, 1845)		Patchwork lampfish	2	1
Myctophiformes	Myctophidae	*Symbolophorus* Bolin & Wisner, 1959	*Symbolophorus* sp.	stet.		11	3
Myctophiformes	Myctophidae	*Taaningichthys* Bolin, 1959	*T.bathyphilus* (Tåning, 1928)		Deepwater lanternfish	1	1
Myctophiformes	Myctophidae	*Taaningichthys* Bolin, 1959	*T.paurolychnus* Davy, 1972			2	1
Myctophiformes	Neoscopelidae	*Scopelengys* Alcock, 1890	*S.tristis* Alcock, 1890		Pacific blackchin	1	1
Stylephoriformes	Stylephoridae	*Stylephorus* Shaw, 1791	*S.chordatus* Shaw, 1791		Tube-eye fish	4	4
Gadiformes			sp.		stet.	1	1
Gadiformes	Melanonidae	*Melanonus* Günther, 1878	*M.gracilis*Norman, 1930 *		Pelagic cod	1	1
Gadiformes	Melanonidae	*Melanonus* Günther, 1878	*M.zugmayeri* Günther, 1878		Arrowtail	2	2
Beryciformes	Melamphaidae	*Melamphaes* Günther, 1864	*Melamphaes* Günther, 1864	stet.		2	1
Beryciformes	Melamphaidae	*Melamphaes* Günther, 1864	*M.microps*Günther, 1878) *			1	1
Beryciformes	Melamphaidae	*Poromitra*Goode & Bean, 1883	*Poromitra* sp.	stet.		1	1
Beryciformes	Melamphaidae	*Poromitra* Goode & Bean, 1883	*P.coronata* (Gilchrist & von Bonde, 1924)			2	1
Beryciformes	Melamphaidae	*Poromitra* Goode & Bean, 1883	*P.megalops* (Lütken, 1878)			21	8
Beryciformes	Melamphaidae	Scopeloberyx Zugmayer, 1911	*Scopeloberyx* sp.	stet.		7	3
Beryciformes	Melamphaidae	*Scopeloberyx* Zugmayer, 1911	*S.opisthopterus* (Parr, 1933)			9	3
Beryciformes	Melamphaidae	*Scopelogadus*Vaillant, 1888	*Scopelogadus* sp.	stet.		105	9
Beryciformes	Melamphaidae	*Scopelogadus* Vaillant, 1888	*S.mizolepis* (Günther, 1878)		Ragged bigscale	2	1
Trachichthyiformes	Anoplogastridae	*Anoplogaster* Günther, 1859	*A.cornuta* (Valenciennes, 1833)		Common fangtooth	1	1
Pleuronectiformes			sp.	stet.	Flatfish	13	1
Lophiiformes	Ceratiidae		sp.	indet.		5	3
Lophiiformes	Ceratiidae	*Ceratias* Krøyer, 1845	*Ceratias* sp.	indet.		2	2
Lophiiformes	Ceratiidae	*Cryptopsaras* Gill, 1883	*C.couesii* Gill, 1883		Triplewart Seadevil	4	3
Lophiiformes	Gigantactinidae	*Gigantactis* Brauer, 1902	*Gigantactis* sp.*	indet.	Whipnose anglerfish	1	1
Lophiiformes	Himantolophidae	*Himantolophus* Reinhardt, 1837	*Himantolophus* sp.		Footballfish	1	1
Lophiiformes	Linophrynidae		sp.*	indet.	Leftvents	2	1
Lophiiformes	Melanocetidae	*Melanocetus* Günther, 1864	*M.johnsonii* Günther, 1864		Humpback anglerfish	19	7
Lophiiformes	Melanocetidae	*Melanocetus* Günther, 1864	*M.murrayi* Günther, 1887		Murray's abyssal anglerfish	2	2
Lophiiformes	Oneirodidae		sp.	stet.	Dreamer	1	1
Lophiiformes	Oneirodidae	*Chaenophryne* Regan, 1925	*Chaenophryne* sp.	stet.		1	1
Tetraodontiformes			sp.	stet.		7	1
Acropomatiformes	Howellidae	*Howella* Ogilby, 1899	*H.atlantica*Post & Quéro, 1991*		Atlantic pelagic basslet	4	3
Acropomatiformes	Howellidae	*Howella* Ogilby, 1899	*H.sherborni* (Norman, 1930)		Sherborn's pelagic bass	3	1

## References

[B7987499] Badcock Julian (1970). The Vertical Distribution of Mesopelagic Fishes Collected on the SOND Cruise. Journal of the Marine Biological Association of the United Kingdom.

[B9875599] Betancur-R Ricardo, Wiley Edward O., Arratia Gloria, Acero Arturo, Bailly Nicolas, Miya Masaki, Lecointre Guillaume, Ortí Guillermo (2017). Phylogenetic classification of bony fishes. BMC Evolutionary Biology.

[B7984067] Boyd Philip W., Claustre Hervé, Levy Marina, Siegel David A., Weber Thomas (2019). Multi-faceted particle pumps drive carbon sequestration in the ocean. Nature.

[B9875725] Brauer A (1901). Über einige von der Valdivia-Expedition gesammelte Tiefseefische und ihre Augen. *Sitzungsberichte der Gesellschaft zur Beförderung der Gesamten Naturwissenschaften zu Marburg*.

[B9875716] Chapman W M (1939). Eleven new species and three new genera of oceanic fishes collected by the International Fisheries Commission from the northeastern Pacific. *Proceedings of the United States National Museum*.

[B7985482] Chavez Francisco P., Messié Monique (2009). A comparison of Eastern Boundary Upwelling Ecosystems. Progress in Oceanography.

[B7985823] Collin Shaun Patrick, Hoskins Robert V, Partridge Julian C (1997). Tubular eyes of deep-sea fishes: a comparative study of retinal topography (part 1 of 2). Brain, Behavior and Evolution.

[B7984162] Davis Alexander L., Sutton Tracey T., Kier William M., Johnsen Sönke (2020). Evidence that eye-facing photophores serve as a reference for counterillumination in an order of deep-sea fishes. Proceedings of the Royal Society B: Biological Sciences.

[B7984180] Davis Alexander L., Thomas Kate N., Goetz Freya E., Robison Bruce H., Johnsen Sönke, Osborn Karen J. (2020). Ultra-black Camouflage in Deep-Sea Fishes. Current Biology.

[B7984144] Denton E. J., Marshall N. B. (2009). The buoyancy of bathypelagic fishes without a gas-filled swimbladder. Journal of the Marine Biological Association of the United Kingdom.

[B8157102] Froese R, Pauly D FishBase. www.fishbase.org.

[B9875752] Günther A (1864). On a new genus of pediculate fish from the Sea of Madeira. *Proceedings of the Zoological Society of London 1864*.

[B9875765] Günther A (1887). Report on the deep-sea fishes collected by H. M. S. Challenger during the years 1873-76. *eport on the Scientific Results of the Voyage of H. M. S. Challenger*.

[B9534781] Hartman S. E., Bett B. J., Durden J. M., Henson S. A., Iveren M., Jeffreys R. M., Horton T., Lampitt R., Gates A. R. (2021). Enduring science: Three decades of observing the Northeast Atlantic from the Porcupine Abyssal Plain Sustained Observatory (PAP-SO. Progress in Oceanography.

[B7987343] Helfman Gene, Collette Bruce B, Facey Douglas E, Bowen Brian W (2009). The diversity of fishes: biology, evolution, and ecology.

[B8157110] Herring P J (1990). RRS Discovery Cruise 195, 05 September-05 October 1990. Photobiology, physiology and distribution of oceanic animals in the tropical North Atlantic.

[B9879922] Horton Tammy, Marsh Leigh, Bett Brian J., Gates Andrew R., Jones Daniel O. B., Benoist Noëlie M. A., Pfeifer Simone, Simon-Lledó Erik, Durden Jennifer M., Vandepitte Leen, Appeltans Ward (2021). Recommendations for the Standardisation of Open Taxonomic Nomenclature for Image-Based Identifications. Frontiers in Marine Science.

[B7984196] Howell Kerry L., Hilário Ana, Allcock A. Louise, Bailey David M., Baker Maria, Clark Malcolm R., Colaço Ana, Copley Jon, Cordes Erik E., Danovaro Roberto, Dissanayake Awantha, Escobar Elva, Esquete Patricia, Gallagher Austin J., Gates Andrew R., Gaudron Sylvie M., German Christopher R., Gjerde Kristina M., Higgs Nicholas D., Le Bris Nadine, Levin Lisa A., Manea Elisabetta, McClain Craig, Menot Lenaick, Mestre Nelia C., Metaxas Anna, Milligan Rosanna J., Muthumbi Agnes W. N., Narayanaswamy Bhavani E., Ramalho Sofia P., Ramirez-Llodra Eva, Robson Laura M., Rogers Alex D., Sellanes Javier, Sigwart Julia D., Sink Kerry, Snelgrove Paul V. R., Stefanoudis Paris V., Sumida Paulo Y., Taylor Michelle L., Thurber Andrew R., Vieira Rui P., Watanabe Hiromi K., Woodall Lucy C., Xavier Joana R. (2020). A blueprint for an inclusive, Global Deep-Sea Ocean Decade Field Program. Frontiers in Marine Science.

[B9875743] Jespersen P, Tåning A V (1926). Mediterranean Sternoptychidae. Report on the Danish Oceanographical Expeditions 1908-10 to the Mediterranean and adjacent seas.

[B7987427] John H Ch, Zelck Clementine (1997). Features, boundaries and connecting mechanisms of the Mauritanian Province exemplified by oceanic fish larvae. Helgoländer Meeresuntersuchungen.

[B10014481] Jordan David Starr, Starks Edwin Chapin (1896). The Fishes of Puget Sound. Proceedings of the California Academy of Sciences.

[B7984123] Munk Ole (1999). The escal photophore of ceratioids (Pisces; Ceratioidei) - a review of structure and function. Acta Zoologica.

[B7987377] Nafpaktitis Basil, Backus Richard H, Craddock James E, Haedrich Richard L, Karnella Charles, Robison Bruce H (1977). Fishes of the western North Atlantic. Part Seven: Order Iniomi (Myctophiformes) Neoscopelidae and Myctophidae and Atlantic Mesopelagic Zoogeography.

[B7987518] Pauly Daniel, Piroddi Chiara, Hood Lincoln, Bailly Nicolas, Chu Elaine, Lam Vicky, Pakhomov Evgeny A, Pshenichnov Leonid K, Radchenko Vladimir I, Palomares Maria Lourdes D (2021). The biology of mesopelagic fishes and their catches (1950–2018) by commercial and experimental fisheries. Journal of Marine Science and Engineering.

[B9858094] Pietsch TW, Van Duzer PJ (1980). Of the family Melanocetidae with the description of a new species from the Eastern North Pacific ocean. Fishery Bulletin.

[B9858321] Pietsch Theodore W. (2009). Oceanic anglerfishes: extraordinary diversity in the deep sea.

[B9858038] Pietsch T W, Kenaley C P Ceratioidei. seadevils, devilfishes, deep-sea anglerfishes. Part of The Tree of Life Web Project.. http://tolweb.org/Ceratioidei/22000.

[B7987368] Pradhan Yaswant, Lavender Samantha J., Hardman-Mountford Nick J., Aiken James (2006). Seasonal and inter-annual variability of chlorophyll-a concentration in the Mauritanian upwelling: Observation of an anomalous event during 1998–1999. Deep Sea Research Part II: Topical Studies in Oceanography.

[B9541435] Priede I. G. (2017). Deep-sea fishes: biology, diversity, ecology and fisheries.

[B7984057] Proud Roland, Handegard Nils Olav, Kloser Rudy J, Cox Martin J, Brierley Andrew S (2018). From siphonophores to deep scattering layers: uncertainty ranges for the estimation of global mesopelagic fish biomass. ICES Journal of Marine Science.

[B8000794] Pugh P (2000). Discovery Collections Midwater Database. National Oceanography Centre, Southampton, UK.

[B8000738] Rainbow Philip S (2005). From natural history to biodiversity: collections of discovery. Archives of natural history.

[B9561003] Reading J., Horton T. (2023). Mauritanian Midwater fish in the Discovery Collections.

[B8001043] Roe Howard SJ, Shale David (1979). A new multiple rectangular midwater trawl (RMT 1+ 8M) and some modifications to the Institute of Oceanographic Sciences' RMT 1+ 8. Marine Biology.

[B9875734] Shaw G (1791). Description of the Stylephoruschordatus a new fish. *The Transactions of the Linnean Society of London*.

[B9858312] Sigovini Marco, Keppel Erica, Tagliapietra Davide (2016). Open Nomenclature in the biodiversity era. Methods in Ecology and Evolution.

[B9552470] Smith R L, Summerhayes C P, Emeis K-C, Angel M V, Smith R L, Scheitzel B (1995). The Physical Processes of Coastal Ocean Upwelling Systems. Upwelling in the ocean: Modern processes and ancient records. Report of the Dahlem Workshop on Upwelling in the Ocean..

[B7987104] Standal Dag, Grimaldo Eduardo (2021). Lost in translation? Practical-and scientific input to the mesopelagic fisheries discourse. Marine Policy.

[B7984086] St. John Michael A., Borja Angel, Chust Guillem, Heath Michael, Grigorov Ivo, Mariani Patrizio, Martin Adrian P., Santos Ricardo S. (2016). A dark hole in our understanding of marine ecosystems and their services: Perspectives from the mesopelagic community. Frontiers in Marine Science.

[B7987509] Sutton Tracy T, Hopkins TL (1996). Trophic ecology of the stomiid (Pisces: Stomiidae) fish assemblage of the eastern Gulf of Mexico: strategies, selectivity and impact of a top mesopelagic predator group. Marine Biology.

[B7987475] Sutton Tracey T, Clark Malcolm R, Dunn Daniel C, Halpin Patrick N, Rogers Alex D, Guinotte John, Bograd Steven J, Angel Martin V, Perez Jose Angel A, Wishner Karen (2017). A global biogeographic classification of the mesopelagic zone. Deep Sea Research Part I: Oceanographic Research Papers.

[B9857966] Sutton Tracey T, Hulley Percy Alexander, Wienerroither Rupert, Zaera-Perez Diana, Paxton John Richard (2020). Identification guide to the mesopelagic fishes of the central and south east Atlantic Ocean.

[B9552462] FAO UN (2020). State of World Fisheries and Aquaculture. Sustainability in Action.

[B9875707] Whitley G P (1943). Ichthyological notes and illustrations (part 2).. Australian Zoologist.

[B9875591] Board WoRMS Editorial World Register of Marine Species. https://www.marinespecies.org.

